# Supplementary material for: Phase-matched high-order harmonic generation in pre-ionized noble gases
Source: Sci Rep. 2022 May 11;12:7715. doi: 10.1038/s41598-022-11313-6 (PMC9095879; doi:10.1038/s41598-022-11313-6)
Supplement: Supplementary file 1 — Supplementary Information. [file 41598_2022_11313_MOESM1_ESM.pdf]

Phase-matched high-order harmonic generation in pre-ionized noble gases: supplemental document

Ondřej Finke, Michal Nevrkla, Lucie Jurkovičová, Martin Albrecht, Alexandr Jančárek, and Jaroslav Nejdl
ELI Beamlines Centre, FZU-Institute of Physics of the Czech Academy of Sciences, Na Slovance 2, 182 21 Prague, Czechia and Czech Technical University in Prague, FNSPE, Břehová 7, 115 19 Prague 1, Czechia

Jan Vábek
ELI Beamlines Centre, FZU-Institute of Physics of the Czech Academy of Sciences, Na Slovance 2, 182 21 Prague, Czechia Czech Technical University in Prague, FNSPE, Břehová 7, 115 19 Prague 1, Czechia and Centre des Lasers Intenses et Applications, Université de Bordeaux-CNRS-CEA, 33405 Talence Cedex, France

Nadezda A. Bobrova
Czech Technical University in Prague, FNSPE, Břehová 7, 115 19 Prague 1, Czechia

Ondřej Hort
ELI Beamlines Centre, FZU-Institute of Physics of the Czech Academy of Sciences, Na Slovance 2, 182 21 Prague, Czechia

Fabrice Catoire
Centre des Lasers Intenses et Applications, Université de Bordeaux-CNRS-CEA, 33405 Talence Cedex, France

Stefan Skupin
Institut Lumière Matière, UMR 5306 Université Lyon 1 - CNRS, Université de Lyon, 69622 Villeurbanne, France

This Supplement of "Phase-matched high-order harmonic generation in pre-ionized noble gases": 1) explains the methodology of the analysis of the numerical data to retrieve the quantities related to the phase of the field, 2) provides more detailed analysis of the various physical models from simple analytical formulas up to a fully numerical treatment.

I. PROCEDURES TO ANALYSE THE NUMERICAL SIMULATIONS

Here, we present the procedure to retrieve the phase of the propagating laser field and the spatial distribution of the coherence length used in Figs. 1 (b), 1 (c) and 5 of the main text. To get an insight, let us start with an analytical model of a complex on-axis field, i.e. we consider an additional geometrical phase, $\phi_{\mathrm{geom.}}$. The field is written as

$$E_{\mathrm{IR}}(z,t) \propto \mathrm{e}^{-\mathbf{i}\left(\omega_0 t - \frac{\omega_0}{c}\int_0^z n(z',t)\,\mathrm{d}z' + \phi_{\mathrm{geom.}}\right)} = \mathrm{e}^{-\mathbf{i}\left(\omega_0 t - \Phi(z,t)\right)}\,. \tag{1}$$

This field induces harmonic response at z_1 written as

$$E^{(1)}_{\mathrm{XUV}}(z_1,t) \propto \mathrm{e}^{-\mathbf{i}\left(q(\omega_0 t - \Phi(z_1,t)) + \phi_{\mathrm{atom},q}(z_1,t)\right)}\,, \tag{2}$$

where the first part of the phase, $q(\omega_0 t - \Phi(z_1,t))$, is imprinted by the driving field, and $\phi_{\mathrm{atom},q}(z_1,t)$ is the phase related to the microscopic generation process of the given harmonic order q. Let us propagate this field infinitesimally to $z_2 = z_1 + \Delta z$, which means only adding $q\omega_0 n_q \Delta z/c$ to the phase. n_q is the refractive index of the harmonic field. If we compare the field generated at z_1 and propagated to z_2 with the field generated at z_2; we have

$$E^{(1)}_{\mathrm{XUV}}(z_2,t) \propto \mathrm{e}^{-\mathbf{i}\left(q(\omega_0 t - \Phi(z_1,t)) + \phi_{\mathrm{atom},q}(z_1,t)\right) + \mathbf{i}\frac{q\omega_0}{c} n_q \Delta z}\,, \tag{3a}$$

$$E^{(2)}_{\mathrm{XUV}}(z_2,t) \propto \mathrm{e}^{-\mathbf{i}\left(q(\omega_0 t - \Phi(z_2,t)) + \phi_{\mathrm{atom},q}(z_2,t)\right)}\,, \tag{3b}$$

respectively. We subtract the phases

$$q\left(\Phi(z_2,t) - \Phi(z_1,t)\right) - \left(\phi_{\mathrm{atom},q}(z_2,t) - \phi_{\mathrm{atom},q}(z_1,t)\right) + \frac{q\omega_0}{c} n_q \Delta z \approx$$
$$\approx \left(\frac{q\omega_0}{c} n_q - q\frac{\partial \Phi(z_1,t)}{\partial z} - \frac{\partial \phi_{\mathrm{atom},q}(z_1,t)}{\partial z}\right)\Delta z = \Delta k_q(z_1,t)\Delta z\,, \tag{4}$$

where we Taylorized the infinitesimal difference of the phases. This procedure provides the phase mismatch, which we denoted $\Delta k_q(z_1, t)$. Because we are within an infinitesimal interval, we can omit the subscript in z_1, the result is a local quantity

$$\Delta k_q(z,t) = \frac{q\omega_0}{c} n_q - q\frac{\partial \Phi(z,t)}{\partial z} - \frac{\partial \phi_{\mathrm{atom},q}(z,t)}{\partial z} \tag{5}$$

with the associated coherence length $L_{\mathrm{coh},q} = |\pi/\Delta k_q|$. An important property is that the result is a function of t. This is justified because the time evolution of the phases is dominant compared to the frequency, which is a natural variable of the refractive-index. We thus evaluate all the quantities at the central frequency of the driver or at the harmonic orders (the aspect of time in the refractive index is discussed in details in [1]).

To evaluate the last term in (5), we need the atomic phase ϕ_{atom}. We use the Saddle-point calculation [2, 3],

$$\phi_q(I) = \phi_q(I_a) + \frac{\partial \phi_q(I_a)}{\partial I}(I - I_a) + \cdots = \phi_q(I_a) - \alpha_q(I_a)(I - I_a) + \cdots \tag{6}$$

in the vicinity of the driver-field intensity I_a. $\alpha_q(I_a)$ is introduced to match the usual expression as used in [4]. The referred works [2–4] show that it is well-approximated by taking only the linear term in the expansion. Using only this term, we obtain

$$\frac{\partial \phi_{\mathrm{atom}}(z,t)}{\partial z} = -\alpha(I(z,t))\frac{\partial I(z,t)}{\partial z}\,. \tag{7}$$

It means this term is locally proportional to the gradient of the intensity.

An advantage of (5) is that it can be used for any complex electric field of the form $E_{\mathrm{IR}} \propto \exp(-\mathbf{i}(\omega_0 t - \Phi(\vec{r}, t))$, even if the decomposition assumed in (1) is not possible. The phase is retrieved after removing fast oscillations as

$$\Phi(\vec{r},t) = \mathrm{Arg}\left(\mathrm{e}^{\mathbf{i}\omega_0 t} E(\vec{r},t)\right)\,. \tag{8}$$

This formula is used to process the numerically computed laser fields $E(\vec{r}, t)$ obtained from the laser-propagation code.

Using the full profile of the laser pulse, Δk_q becomes fully spatio-temporal quantity in $(t, \vec{r})$. We will need to choose some cuts to visualise it. To analyse the spatial dependence, we fix time in the peak of the pulse, where the maximal HHG production is expected; this procedure agrees with the one used in [5]. Note, that the homogeneous pre-ionization is only a constant shift in $\Delta k_q(z, t)$, the choice of the reference time is then fully consistent across various initial conditions.

Finally, we return to the physical interpretation of (5). It is rewritten with the help of the relation between indexes of refraction as $n \approx 1 + \chi_{\mathrm{disp.}}/2 + \chi_{\mathrm{plasma}}/2$ as

$$\Delta k_q = \underbrace{\frac{q\omega_0}{2c}\left(\chi_{\mathrm{disp.},q} - \chi_{\mathrm{disp.,IR}}\right)}_{\Delta k_{\mathrm{disp.}}} \underbrace{- \frac{q\omega_0}{2c}\chi_{\mathrm{plasma,IR}}}_{\Delta k_{\mathrm{plasma}}} \underbrace{-q\frac{\partial \phi_{\mathrm{geom.}}}{\partial z}}_{\Delta k_{\mathrm{geom.}}} \underbrace{-\frac{\partial \phi_{\mathrm{atom},q}}{\partial z}}_{\Delta k_{\mathrm{atom}}}\,. \tag{9}$$

The plasma effect is not taken into account for the XUV-field as the induced phase is negligible in the spectral range under consideration. This is exactly the usual formula used to study phase-matching from [6]. We stress here that all the effects are present for our numerical field. The stable intensity region of the interest means small gradients of intensity and geometrical phase. The role of $\Delta k_{\mathrm{geom.}}$ and k_{atom} is thus minor in this region. The details of this assumption follow in section II by comparing the full-numerical and analytical models.

II. BENCHMARKING MODELS: PHASE MISMATCH FROM IONISATION RATIO η

We have used the analytical model of the mismatch, Eq. (3) in the main manuscript, for a basic picture of the problem; its only variable is the ionization degree η, which is a much simpler quantity than the phase (8). Additionally, we have introduced an *ab initio* numerical method to compute the same quantity in the previous paragraph (5). Natural questions are: How do these two compare? Do the analytical and numerical models agree quantitatively? In other words, we now benchmark these models.

We start with the phase mismatches shown in Fig. 1(b,c), which were obtained from the numerical analysis (5). Figure 1 compares these values with the analytical model, where only η is taken from the numerical propagation. The two models agree well in the stable region without the pre-ionization (panels (a) and (b)). The scale is also

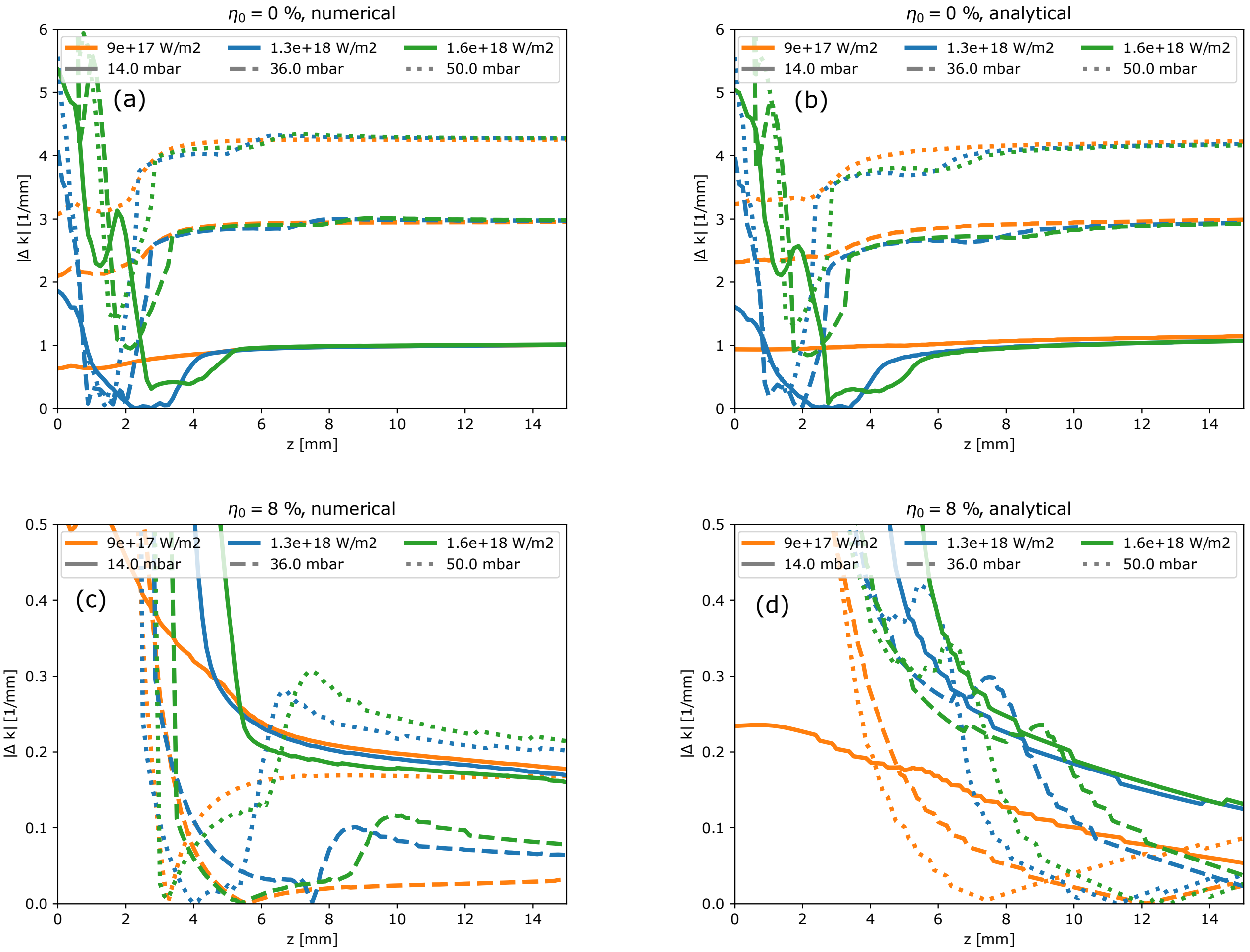

FIG. 1. The comparison of the two models for computing Δk (numerical based on (5) and analytical based on Eq. (3)). Left panels show the results of the complete calculation already discussed in Fig. 1(b,c), the right panels are their counterparts computed from the analytic approach using ionization ratios at the peak of the pulse obtained from the numerical laser propagation.

correct with the 8 % pre-ionization, but the results differ more (panels (c) and (d)). This is not surprising, because the differences from other effects – the beam geometry and the microscopic phase of the dipole – are at play once the dominant effect of the ionization is removed. However, these differences remain within the range that is still favorable for phase-matching. In summary, these results confirm that the simple model describes well the dominant effect and may be used for an optimization procedure.

We compare also the maps of the coherence length shown in Fig. 5 of the main article. The result is shown in Fig. 2. The overall scale is correct and the models agree well in the most important region: near the axis in the stabilized region II. There are differences in the off-axis region ($r > 50$ μm), where intensity gradients make the atomic dipole phase more significant. Because η is the only variable, the constant L_{coh} for $r > 50$ μm in the right panels shows that there is no ionisation. It means that the dominant region for the harmonic generation is $r < 50$ μm. The non-trivial evolution of the phase outside this region for the numerical model is related to the transverse gradient of the field.

We recall that these maps are only snapshots in the peak of the pulse. We have also investigated the change with different time near by the peak of the field envelope. The main trend, however, remained stable. Since the non-linear propagation is a complex process, a snapshot in time gives only a strong indication that an optimal L_{coh} would lead to a large gain. The benchmark of this is the fully numerical model.

In conclusion, we have proven that the analytical model works well to describe the dominant effect – the ionization – in the spatial region relevant for HHG. Next, it is also a good benchmark of the code: Although the initial electric field and plasma density come both from the same propagation code, the treatment of the phase is then completely *independent*. In the first case, the phase mismatch is obtained from the numerical differentiation of the phase of the

numerical field. In the second case, it is only the degree of ionization η as a result of the propagation code used in the analytical model. Finally, all these results are consistent and agree with the fully numerical model from the main manuscript. It proves the ability of the analytical model to be predictive for the physics investigated.

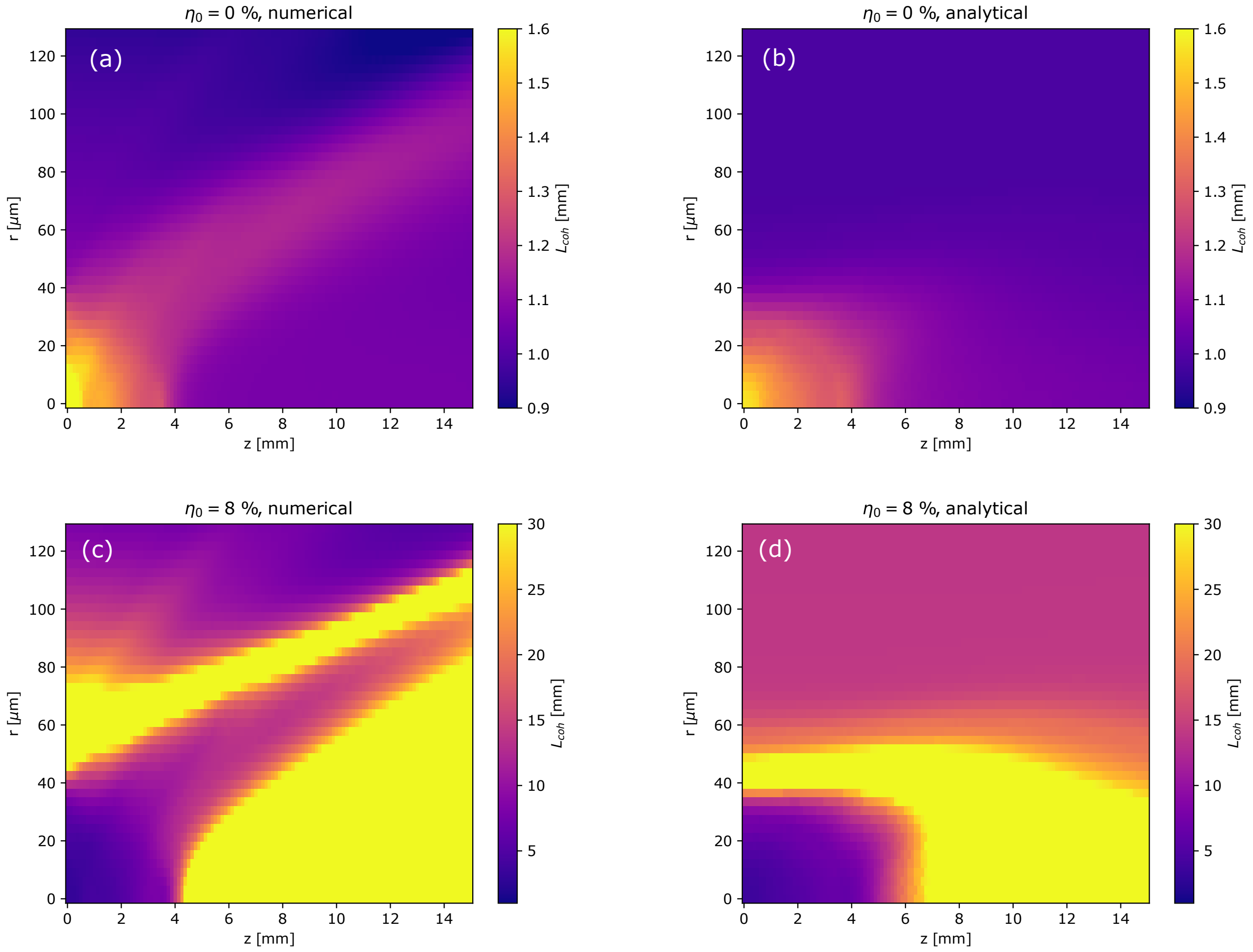

FIG. 2. The comparison of the two models for computing L_{coh}. Left panels show the results presented in Fig. 5, the right panels are their counterparts computed from the analytic approach, similarly to Fig. 1.

[1] M. A. Khokhlova and V. V. Strelkov, Highly efficient XUV generation via high-order frequency mixing, New Journal of Physics **22**, 093030 (2020).

[2] M. B. Gaarde, A. L'Huillier, and M. Lewenstein, Theory of high-order sum and difference frequency mixing in a strong bichromatic laser field, Phys. Rev. A **54**, 4236 (1996).

[3] M. B. Gaarde, F. Salin, E. Constant, P. Balcou, K. J. Schafer, K. C. Kulander, and A. L'Huillier, Spatiotemporal separation of high harmonic radiation into two quantum path components, Phys. Rev. A **59**, 1367 (1999).

[4] F. Catoire, A. Ferré, O. Hort, A. Dubrouil, L. Quintard, D. Descamps, S. Petit, F. Burgy, E. Mével, Y. Mairesse, and E. Constant, Complex structure of spatially resolved high-order-harmonic spectra, Phys. Rev. A **94**, 063401 (2016).

[5] D. E. Rivas, B. Major, M. Weidman, W. Helml, G. Marcus, R. Kienberger, D. Charalambidis, P. Tzallas, E. Balogh, K. Kovács, V. Tosa, B. Bergues, K. Varjú, and L. Veisz, Propagation-enhanced generation of intense high-harmonic continua in the 100-ev spectral region, Optica **5**, 1283 (2018).

[6] C. Delfin, C. Altucci, F. D. Filippo, C. de Lisio, M. B. Gaarde, A. L'Huillier, L. Roos, and C.-G. Wahlström, Influence of the medium length on high-order harmonic generation, Journal of Physics B: Atomic, Molecular and Optical Physics **32**, 5397 (1999).
